# Barriers to the Large-Scale Adoption of a COVID-19 Contact Tracing App in Germany: Survey Study

**DOI:** 10.2196/23362

**Published:** 2021-03-02

**Authors:** Annelies G Blom, Alexander Wenz, Carina Cornesse, Tobias Rettig, Marina Fikel, Sabine Friedel, Katja Möhring, Elias Naumann, Maximiliane Reifenscheid, Ulrich Krieger

**Affiliations:** 1 Collaborative Research Center 884 “Political Economy of Reforms” University of Mannheim Mannheim Germany; 2 School of Social Sciences University of Mannheim Mannheim Germany

**Keywords:** digital health, mobile health, smartphone, mobile phone, app, digital technology, contact tracing, coronavirus, COVID-19, survey

## Abstract

**Background:**

During the COVID-19 pandemic, one way to reduce further transmissions of SARS-CoV-2 is the widespread use of contact tracing apps. Such apps keep track of proximity contacts and warn contacts of persons who tested positive for an infection.

**Objective:**

In this study, we analyzed potential barriers to the large-scale adoption of the official contact tracing app that was introduced in Germany on June 16, 2020.

**Methods:**

Survey data were collected from 3276 adults during the week the app was introduced using an offline-recruited, probability-based online panel of the general adult population in Germany.

**Results:**

We estimate that 81% of the population aged 18 to 77 years possess the devices and ability to install the official app and that 35% are also willing to install and use it. Potential spreaders show high access to devices required to install the app (92%) and high ability to install the app (91%) but low willingness (31%) to correctly adopt the app, whereas for vulnerable groups, the main barrier is access (62%).

**Conclusions:**

The findings suggest a pessimistic view on the effectiveness of app-based contact tracing to contain the COVID-19 pandemic. We recommend targeting information campaigns at groups with a high potential to spread the virus but who are unwilling to install and correctly use the app, in particular men and those aged between 30 and 59 years. In addition, vulnerable groups, in particular older individuals and those in lower-income households, may be provided with equipment and support to overcome their barriers to app adoption.

## Introduction

Since the outbreak of COVID-19, millions of people worldwide have been infected with SARS-CoV-2 [1]. In the absence of an effective vaccine or cure, societies across the globe are testing various combinations of measures to contain the spread of the virus [2]. Many countries have introduced lockdowns to reduce the number of new infections to a level that allows national health systems to treat all patients effectively despite the additional influx of seriously ill people [3].

While lockdowns have proven effective at reducing the spread of the virus, they have a major impact on the economy and social life [4,5]. A less economically damaging measure is contact tracing, where persons who have been in close proximity of someone known to be infected are quarantined until they can be confirmed not infected (ie, tested negative) or, if confirmed infected (ie, tested positive), until they are not contagious anymore. In Germany, this task has been performed by officials in local public health departments, who through personal conversations with infected persons, have been collecting proximity contacts to inform them of their potential infection and to implement quarantines [6].

To grant some relief to this labor-intensive system and to account for regionally strewn sudden new outbreaks, scientists have been discussing app-based contact tracing as a supplementary measure [7]. Once installed on a smartphone, a contact tracing app warns users when they have been in close contact with an infected person and may advise them to go into quarantine and get tested for infection. Thus, if adopted widely, apps may allow for a more efficient tracing of infection chains.

On June 16, 2020, 141 days after the first diagnosis of COVID-19 in Germany [8], the federal government and the Robert Koch Institute (RKI) (ie, the German center for disease control and prevention) launched their official COVID-19 contact tracing app [9,10]. Simulations estimate that for the app to contain the epidemic, at least 56% of a country’s population needs to use the app and comply with the app’s recommendations [11], although lower uptake rates are also effective in reducing the number of infections [12]. This paper examines to what extent this goal is likely to be achieved in Germany by providing answers to the following research question: What proportions of the general population aged 18 to 77 years in Germany (1) have access to the devices required to install the official contact tracing app, (2) are able to install it, and (3) are willing to install the app, use it, and act according to its recommendations?

Our predictions show that the adoption rate of 56% needed to contain the epidemic will be missed by a considerable margin. However, contact tracing apps may still be effective if specific subgroups adopted them at a higher rate. In particular, if a high proportion of persons who are frequently in contact with persons outside their household (ie, potential spreaders) adopted the app, its spread may be significantly curbed. In a similar vein, if a high proportion of persons who are likely to get severely ill from the disease (ie, vulnerable groups) adopted the app, health workers may be able to treat them early on and, thus, decrease the impact of COVID-19. Therefore, we investigate adoption rates among these two population subgroups by asking the following research questions:

1. What proportions of potential spreaders (1) have access to the devices required to install the official contact tracing app, (2) are able to install it, and (3) are willing to install the app, use it, and act according to its recommendations?
2. What proportions of persons with high vulnerability to a serious infection (1) have access to the devices required to install the official contact tracing app, (2) are able to install it, and (3) are willing to install the app, use it, and act according to its recommendations?

The official COVID-19 contact tracing app in Germany, the Corona-Warn-App, can be downloaded from the Apple App Store or Google Play free of charge and installed on iPhones, with iOS version 13.5 or higher, and Android smartphones, with Android version 6.0 or higher [9,10]. The app can be installed by the same person on multiple devices. Once installed, the app detects other app users in proximity by exchanging encrypted ID numbers between devices using Bluetooth Low Energy technology. The ID numbers change constantly and are stored locally on the device, relying on a decentralized approach for data storage. The user’s geolocation is not tracked. The app automatically informs users when they have been in contact with someone confirmed infected with SARS-CoV-2 and provides behavioral recommendations, including domestic quarantine and tests for SARS-CoV-2. The identity of the person using the app remains anonymous. An app user with a positive test result can enter this result into the Corona-Warn-App. By doing so, all proximity contacts are automatically notified of their own potential infection. Users can deactivate and reactivate the COVID-19 exposure notifications at any time or can completely uninstall the app. Using the Corona-Warn-App is voluntary and meets the European Union General Data Protection Regulation [13,14].

There are several potential barriers that may prevent people from using an app [15,16]. An initial barrier is access to a smartphone capable of installing the desired app and access to the internet [17,18]. In the case of the Corona-Warn-App, persons additionally need a smartphone with an iOS or Android operating system [9,10]. Among smartphone users with compatible devices, a second barrier is their ability to carry out the tasks required to operate the app [19]. The Corona-Warn-App requires the user to have the ability to download and install the app and to handle Bluetooth [9,10]. A final potential barrier is a person’s willingness to use the contact tracing app. A key correlate of this barrier in Germany seems to be privacy concerns regarding the sharing of personal data and distrust in unfamiliar technology and processes running in the background [20,21].

The effectiveness of contact tracing apps not only hinges on access, ability, and willingness to use such an app, but also on *how* individuals use the app. People need to carry their smartphone with them throughout the day, regularly recharge the smartphone batteries, keep their smartphone turned on, and keep the contact tracing feature activated so that the app can detect proximity contacts at all times. For some activities, however, people usually do not take their phone with them; for example, while exercising. As a result, the contacts during these periods are not being tracked. In addition, app-based contact tracing is subject to technical limitations, such as Bluetooth-based measurement errors, which may cause errors in the contacts detected [22].

COVID-19 exposure apps have been developed in many countries [23-25]. The MIT Technology Review’s Covid Tracing Tracker currently lists 47 countries with available or soon-to-be available contact tracing apps [23], yet installation rates across countries are low. For example, there were only 22.4 million app downloads in Germany as of November 12, 2020, around 5 months after its introduction, compared to a population of 83.2 million [10], even though an early Oxford-led study suggested high support for contact tracing apps of 74.8% across five countries: France, Germany, Italy, the United Kingdom, and the United States [26]. However, the Oxford study comes with a major caveat: the predictions were based on nonprobability online samples, which are known to be self-selective and severely overrepresent technologically interested persons; thus, they do not accurately represent likely behaviors in those countries’ populations [27]. The selective nature of the data may, therefore, explain the discrepancy between the high support for the apps in the Oxford study and observed installation rates.

## Methods

### Data

To allow for timely and accurate population predictions of the adoption of the Corona-Warn-App, we based our analyses on data collected close to the launch of the app and on a probability sample of the general population aged 18 to 77 years. In this section, we describe key aspects of our data collection according to the Checklist for Reporting Results of Internet E-Surveys (CHERRIES) [28].

The data were collected in the Mannheim Corona Study (MCS) [29]. The MCS was implemented within the German Internet Panel (GIP), a long-standing, offline-recruited, probability-based online panel of the general adult population in Germany. GIP sample members were recruited in 2012, 2014, and 2018. The 2012 and 2014 recruitments were based on area probability samples with full address listings and face-to-face recruitment interviews [30]. Persons in households without internet and/or computer access were provided with user-friendly devices, internet connections, and/or information technology support to enable their participation in the panel [31]. The 2018 recruitment was based on a probability sample drawn from municipal population registers and initial postal invitations. Sample members were informed about the scope of the study, the investigator, as well as how their data would be stored, and they consented to their participation and data storage.

A subsample of the GIP was invited to participate in the MCS that was conducted for 16 weeks from March 20 to July 10, 2020. The MCS was fielded with a rotating daily panel design. Each week the same MCS sample members were invited via email to participate in the study on the same day of the week and were re-interviewed on a variety of social, psychological, and economic topics [29]. They were sent a personalized link to the survey or were able to log in to the study website using their username and password to access the survey. Upon survey completion, respondents received an incentive of €2 (US $2.40). Our study on the Corona-Warn-App, which was launched on June 16, 2020, was implemented within the MCS in week 13 (ie, June 12 to 19, 2020). The questionnaire from that week contained 44 pages, with one item per page. Respondents were able to change their answers using a *back* button on most pages. Prior to fielding the study, the usability and technical functionality of the questionnaire was tested. A total of 5427 persons, aged 18 to 77 years, were invited to participate in our study, of which 3276 responded (60.4%).

To correct for a potential overrepresentation of persons with higher digital affinity [32], all predictions were weighted with a two-stage weighting procedure. Our weighting accounted for potential coverage, sampling, and nonresponse biases of the online data collection [33,34]. At the first stage, we estimated a response propensity weight, which projected the characteristics of the MCS respondents to the GIP recruitment samples. The weighting characteristics for the 2012 and 2014 samples included computer and internet access within the household; weighting characteristics for the 2018 sample included frequency of internet use, intensity of internet use, computer use, smartphone use, tablet use, and importance of up-to-date technology. At the second stage, we estimated a raking weight, which extrapolated the characteristics of the MCS respondents to the general population according to the Mikrozensus, that is, official statistics provided by the German Federal Statistical Office [35]. The weighting characteristics included age, gender, marital status, highest level of education, household size, and federal state. Missing values on the weighting variables were imputed with a chained-equations algorithm [36]. The final weight was trimmed for values greater than 4 and values less than 0.25. Despite the weighting procedure, our analyses were still likely to overestimate the app adoption rate in the general population, which we further address in the Discussion section.

### Measures

#### Access, Ability, and Willingness

We measured adoption rates and potential barriers to adoption in sequential sets of survey questions and estimated (1) the population’s access to and use of compatible smartphones, (2) their ability to install and correctly use the app, and (3) their willingness to adopt the app and act according to its instructions.

Through three questions, we estimated people’s access: “Do you personally use a smartphone?”; if *yes*, “Which of the following types best describes your smartphone?” and “How often do you carry your smartphone with you when you leave the house?” (see Multimedia Appendix 1). We defined persons as having access to the app if they own an iPhone or Android phone and carry it with them at least most of the time when they leave the house. We did not differentiate between operating system versions and were, thus, likely to overestimate access to the app. However, research about the distribution of operating system versions installed on smartphones in Germany suggests that the large majority of Android smartphones and iPhones have the version installed that is required for the Corona-Warn-App to work [37,38].

People’s ability to use the app was measured through four questions: “Do you know how to install an app, i.e. an additional program, on your smartphone?”; if *no* or *not sure*, “Do you know anyone who could help you with installing the Corona-Warn-App on your smartphone, e.g. family, friends, or neighbors?”; “Do you know how to activate Bluetooth on your smartphone?”; and if *no* or *not sure*, “Do you know anyone who could help you with the activation of Bluetooth on your smartphone, e.g. family, friends, or neighbors?” (see Multimedia Appendix 1). A limitation of the two questions about the potential help of family, friends, or neighbors is that they do not differentiate between the usual situation before the COVID-19 lockdown measures came into effect and the present situation: while some individuals may generally know plenty of people who can assist them with technology-related issues, they may not be able to meet these people due to the lockdown measures. However, since infection rates fell in June 2020 and the lockdown measures were gradually lifted, this limitation likely ceased to affect adoption rates during the summer. We defined persons as able to use the app if they know how to install an app or have someone who can help them with it, and if they know how to activate Bluetooth on their smartphone or have someone who can help them with it. Since access is a necessary condition for being able to install the Corona-Warn-App on a smartphone, persons who were defined as not having access were also defined as not being able to use the app.

Finally, we measured people’s willingness to correctly use the app through four questions: “Would you install the official Corona-Warn-App on your smartphone when it is available?”; if at least *probably not install*, “Would you follow the request of the Corona-Warn-App and go into domestic quarantine as a precaution?”; “Would you comply with the request of the Corona-Warn-App and get tested for the virus?”; and “Would you enter the test result into the Corona-Warn-App if you were tested positive for the virus?” (see Multimedia Appendix 1). We defined persons as willing to correctly use the app if they are probably or definitely willing to install the app, if they are probably or definitely willing to quarantine if requested, if they are probably or definitely willing to get tested if requested, and if they are probably or definitely willing to enter their own test result into the app if they were tested positive. Since access and ability are necessary conditions for being willing to install the Corona-Warn-App on their smartphone, persons who were defined as not having access or not being able to use the app were also defined as not being willing to correctly use the app.

#### Potential to Spread SARS-CoV-2 and Potential to Be at Risk of COVID-19

Two variables from the MCS and GIP data collection classified persons according to their potential for spreading the virus: number of social contacts within the past 7 days and employment situation, both collected from the MCS in week 13 (see Multimedia Appendix 1). The resulting variable has the following categories:

1. Met socially with other persons several times in the past 7 days and worked full time outside the home.
2. Met socially with other persons several times in the past 7 days but did not work full time or did not work outside the home.
3. Met socially with other persons once or less often in the past 7 days but worked full time outside the home.
4. Met socially with other persons once or less often in the past 7 days and did not work full time or did not work outside the home.

In addition, two variables from the MCS and GIP data collection classified persons according to their potential for being vulnerable to a serious infection: being aged 60 to 77 years, collected from the GIP, and having any health condition that, according to the RKI, may be correlated with an increased risk, collected from the MCS in week 13 (see Multimedia Appendix 1). The resulting variable has the following categories:

1. Aged 60 to 77 years and with at-risk health conditions.
2. Aged 60 to 77 years but without at-risk health conditions.
3. Aged 18 to 59 years but with at-risk health conditions.
4. Aged 18 to 59 years and without at-risk health conditions.

Although participants were not required to respond to all questions in the MCS survey, the amount of missing data was low for frequency of social contacts (8 missing values), work outside home (1 missing value), and health condition (5 missing values).

### Analytical Strategy

First, we reported overall rates of Corona-Warn-App adoption, distinguishing the three levels of potential barriers: access, ability, and willingness. Subsequently, we estimated separate adoption rates by the potential to spread SARS-CoV-2 and the potential to be vulnerable to COVID-19. All estimations were weighted as described above to enable reliable population predictions. Adoption rates across subgroups were reported by means of the predicted probabilities of a logistic regression, not including any covariates. Using the margins command in Stata 16.0 (StataCorp LLC), predicted probabilities were computed to conduct chi-square tests of differences in adoption rates across subgroups. Finally, we examined whether the introduction of the Corona-Warn-App during our data collection period influenced people’s willingness to install and use the app. For this purpose, we estimated a logistic regression for willingness on a dummy variable identifying whether our data were collected before or after the publication of the Corona-Warn-App, controlling for key sociodemographic characteristics (see Multimedia Appendix 1).

## Results

**Figure 1:**
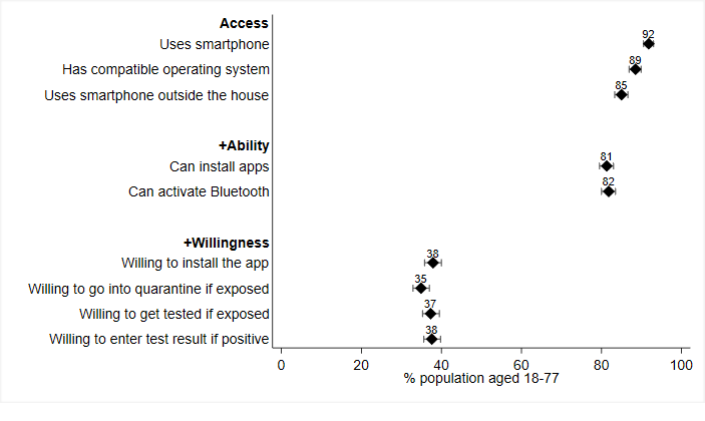
Predicted adoption rates by access, ability, and willingness (N=3276). Error bars represent 95% CI.

For the overall rate of adoption of the Corona-Warn-App, we estimated that 37.9% of the population in Germany aged 18 to 77 years have access to, are able to, and are willing to install the app (see Figure 1 and Multimedia Appendix 2). Asked whether they would be willing to go into domestic quarantine and get tested when requested to do so by the app, these rates reduce to 34.9% and 37.3%, respectively. If tested positive, 37.6% of the population aged 18 to 77 years would be willing to enter the test result into the app.

Whereas a lack of willingness is the foremost barrier to app adoption, access also plays a considerable role. Only 91.8% of the population aged 18 to 77 years uses a smartphone, 88.5% uses one with a compatible operating system, and 85.0% carries it with them most or all of the time when outside the house. An inability to install apps and handle Bluetooth further reduces potential adoption rates to 81.3% and 81.8%, respectively.

**Figure 2:**
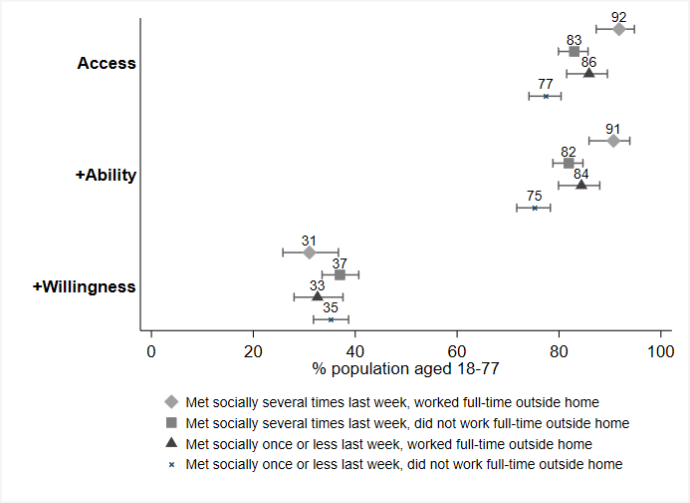
Predicted adoption rates by potential to spread SARS-CoV-2 (access: N=3267; ability: N=3267; willingness: N=3266). Error bars represent 95% CI.

**Figure 3:**
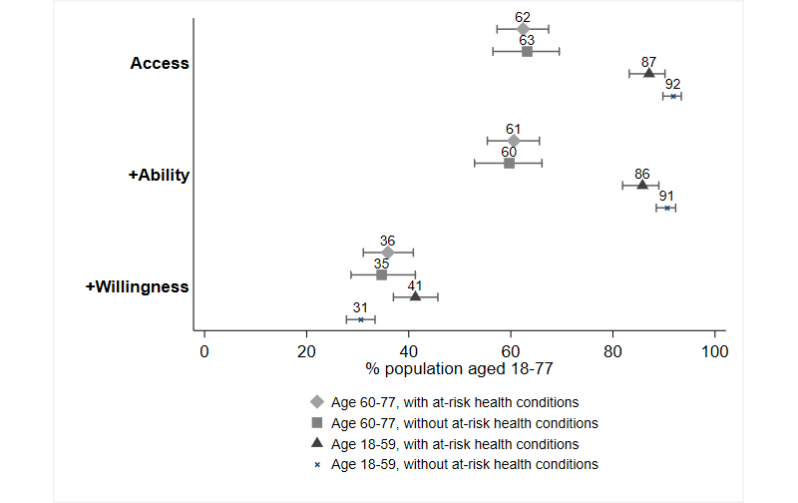
Predicted adoption rates by potential vulnerability to COVID-19 (access: N=3270; ability: N=3270; willingness: N=3269). Error bars represent 95% CI.

Next, we examined whether higher adoption rates were achieved among the relevant subgroups of potential spreaders (see Figure 2 and Multimedia Appendix 3) and the potentially vulnerable (see Figure 3 and Multimedia Appendix 4).

Persons with a high potential to spread the virus (ie, met socially several times last week and worked full time outside the home: 91.8%) are significantly more likely to have access than those with a medium potential to spread the virus (ie, met socially several times last week and did not work full time outside the home: 83.0%; and met socially once or less often last week and worked full time outside the home: 85.9%). Persons with a medium potential to spread the virus are, in turn, significantly more likely to have access than persons with a low potential to spread the virus (ie, met socially once or less often last week and did not work full time outside the home: 77.4%). The same pattern of significant group differences was found for ability (90.7% vs 81.9% and 84.4% vs 75.2%). However, in predicting the overall adoption rates (ie, access + ability + willingness), we did not find any significant group differences (ie, met socially several times last week and worked full time outside the home: 31.0%; met socially several times last week and did not work full time outside the home: 37.0%; met socially once or less last week and worked full time outside the home: 32.6%; and met socially once or less last week and did not work full time outside the home: 35.2%). In fact, the pattern does not deliver support for the hope that the contact tracing app may be more effective in the subgroup with a high potential to spread the virus, with similarly low overall adoption rates for those with a high potential to spread SARS-CoV-2 compared to those with a medium or low potential.

When examining the characteristics of those with a high potential to spread SARS-CoV-2 but unwilling to install and correctly use the Corona-Warn-App, we found that the large majority (68%) are between the ages of 30 and 59 years, with an additional 22% between 18 and 29 years and 10% between 60 and 77 years. Furthermore, most of these individuals are male (67%), with an intermediate (42%) or higher education (40%) as opposed to a lower education (18%). Only 10% feel personally threatened by COVID-19, while a majority (65%) think that the economic damage of the measures taken by governments to fight the pandemic is greater than their benefit for society. Interestingly, privacy concerns do not seem to be the driving factors that influence their decision to not adopt the app, since only 13% indicated they are very concerned about their privacy.

Regarding potential vulnerability, we observed an age effect on access and ability (see Figure 3 and Multimedia Appendix 4). The older age groups (ie, aged 60-77 years, with at-risk health conditions; and aged 60-77 years, without at-risk health conditions) are significantly less likely than younger age groups (ie, aged 18-59 years, with at-risk health conditions; and aged 18-59 years, without at-risk health conditions) to use a compatible smartphone (62.4% and 63.2% vs 87.1% and 91.8%) and to be able to install and use the app (60.6% and 59.7% vs 85.8% and 90.6%), with very large differences independent of at-risk health conditions. In predicting the overall adoption rates (ie, access + ability + willingness), we did not find any consistent significant differences across vulnerability groups (ie, aged 60-77 years, with at-risk health conditions: 35.9%; aged 60-77 years, without at-risk health conditions: 34.7%; aged 18-59 years, with at-risk health conditions: 41.3%; and aged 18-59 years, without at-risk health conditions: 30.6%).

When examining the characteristics of those with a high vulnerability to COVID-19 who do not have access and are unable to use the app, we found that the majority (43%) are older than 70 years, with an additional 28% between 60 and 64 years and 29% between 65 and 69 years. Most of these individuals are living in lower-income households, with a monthly net income between €0 (US $0) and €1999 (US $2414) (41%) or between €2000 (US $2415) and €2999 (US $3623) (39%), as opposed to those living in higher-income households (ie, between €3000 [US $3624] and €3999 [US $4830]: 11%; and €4000+ [US $4831+]: 10%).

Finally, when examining whether persons who were interviewed before the introduction of the Corona-Warn-App showed different adoption rates than persons interviewed after the app launch, we found no significant differences (see Multimedia Appendix 5).

## Discussion

The official contact tracing app by the German federal government and the center for disease control and prevention, RKI, was introduced on June 16, 2020. The Corona-Warn-App was heavily advertised by government officials and health representatives as an effective way to contain the spread of SARS-CoV-2. According to epidemiological models, however, 56% of the population needs to adopt the app for it to contain the epidemic [11].

Our study shows that the 56% target mark will likely be missed by a considerable margin. For the population aged 18 to 77 years, our estimations predict an overall adoption rate of 34.7%. The largest barrier is people’s willingness to install and correctly use the app; however, access to a compatible smartphone and the ability to install the app also play roles. Given the age groups covered in our study, we consider this an optimistic estimate. For cohorts aged 78 years and over and children, the adoption rates are likely considerably lower.

Persons with the highest potential to spread the virus (ie, with frequent social and work contacts) are more likely to have access and the ability to use the app (90.7%) than the average in the population aged 18 to 77 years (81.0%). Overall, persons with a high potential to spread the virus are no more likely to adopt the app than persons with fewer social and work interactions.

Persons at risk to fall seriously ill or die from an infection (ie, those aged 60 to 77 years with at-risk health conditions) have significantly reduced access and ability to use the app (60.6%) compared to the average in the population aged 18 to 77 years (81.0%). Those who can use the app, however, are overwhelmingly willing to do so. As a consequence, persons with high vulnerability to COVID-19 are equally likely to adopt the app as are less vulnerable groups.

Overall, the findings imply a pessimistic view on the effectiveness of app-based contact tracing to contain the COVID-19 pandemic in Germany, with low adoption rates in the general population and issues of selectivity across subgroups as noted by Klingwort and Schnell [22]. In addition to low uptake in the general population, vulnerable groups who would benefit from an efficient contact tracing approach have limited smartphone coverage and limited ability to use the app. Furthermore, those with a high potential to spread SARS-CoV-2 who would have the necessary devices and abilities to install the app are predominantly unwilling to do so. Even though, as Hinch et al [11] pointed out, uptake rates of contact tracing apps lower than the 56% target may still contribute to a reduction in the number of infections, Germany will miss the 56% target by a huge margin and would do well investing in additional routes of tracing potentially infected individuals.

Our study was conducted during the week the Corona-Warn-App was introduced in Germany. This enabled us to implement a questionnaire that considers all technological and data privacy specifications of the actual app. The findings also allow us to formulate actionable policy recommendations. First, we recommend targeting information campaigns at groups with a high potential to spread the virus but who are unwilling to install and correctly use the Corona-Warn-App, in particular men and those aged between 30 and 59 years, to encourage them to adopt the app. Our second recommendation is to invest further resources to provide vulnerable groups of the population, in particular older individuals and those in lower-income households, with the necessary devices and assistance to overcome their specific barriers to app adoption.

This study is not free from limitations. First, the data were collected from an online panel. Although individuals without computer or internet access were provided with the necessary equipment and support, and weights were used in all analyses to correct for coverage and nonresponse biases, we cannot rule out that the data still overrepresent individuals with an interest in technology. When invited to the MCS, panel members had already completed online surveys over the course of at least 2 years. They are, thus, more likely to be interested in digital technologies, such as the Corona-Warn-App, than their counterparts who dropped out of the online panel.

Second, we can expect panel members who agreed to participate in the MCS and be interviewed every week on topics related to the COVID-19 pandemic to be more interested in contributing to a better understanding of the social impacts of the pandemic and possibly be more concerned than the average citizen. Such traits may also affect their willingness to install a contact tracing app.

Third, our analyses are limited to individuals aged between 18 and 77 years. We are, thus, missing sizable population groups: those aged 0 to 10 years make up 10% of the general population in Germany, those aged 11 to 17 years constitute 6%, and those aged 78 years or older make up 9% [39]. The youngest age group might be disregarded in an estimation of the effectiveness of the Corona-Warn-App since they predominantly move within small, defined social circles and are unlikely to carry smartphones with them at all times. The age group of 11 to 17 years is likely to have a high potential to spread SARS-CoV-2 but possibly low interest in adopting the Corona-Warn-App, whereas the oldest age group is highly vulnerable to COVID-19 and is also likely to have low app adoption rates because of limited smartphone access. As a result of all of these limitations, our predicted adoption rates are an optimistic view of the situation. True values in the general population are likely to paint an even more pessimistic reality.

Fourth, our study is based on reported hypothetical behavior rather than actual behavior. Although hypothetical measures of willingness to install an app are subjective and may be subject to various response errors, such as social desirability or recall errors [40], these measures were shown to be correlated with actual behavior in previous studies [15,16,20,41]. In weeks 14 to 16 of the MCS, fielded between June 19 and July 10, 2020, we also collected data about whether people installed the Corona-Warn-App on their smartphone. The results suggest that the actual installation rate is very similar to the app adoption rate estimated in this paper: by July 10, 2020, almost 1 month after the app was introduced, 36% (95% CI 34%-38%) of the population between 18 and 77 years had installed the app, 55% (95% CI 53%-58%) had not installed the app, 1% (95% CI 1%-2%) had installed the app but had uninstalled it since then, and 7% (95% CI 6%-8%) did not use a smartphone. We also compared the responses about hypothetical and actual willingness to install the app among 2877 survey respondents who completed the questions both in week 13 and week 16 of the MCS survey, combining the categories *app not installed*; *app installed, but uninstalled since then*; and *don’t use a smartphone*. The results show a correlation of *r*=0.6 (*P*<.001), which gives us confidence that the hypothetical measures used in this paper are rather accurate.

A potential avenue of future research would be to study whether the use of such a contact tracing app changes people’s behavior. After the installation, individuals’ behavior may become riskier (eg, less compliant toward social distancing measures), since the app may give them a feeling of security. If such a behavior is prevalent in the population, this may further reduce the effectiveness of app-based contact tracing.

Regarding data availability, the GIP data used in the analyses of this article are freely available as part of the GIP Scientific Use Files. They can be requested from the GESIS Data Archive for the Social Sciences (GESIS-DAS) [42]. The MCS data are envisioned to be published as Scientific Use Files by the end of 2021 at the latest. Until then, these data can be accessed at the Onsite Data Access facilities of the GIP Secure Data Center located at the Collaborative Research Center *Political Economy of Reforms* (SFB 884), University of Mannheim, B6 30-32, Mannheim, Germany. Researchers wishing to make use of the Onsite Data Access facilities may contact secretary@reforms.uni-mannheim.de. Researchers wishing to get access to the analysis code may contact the corresponding author.

## Appendix

Multimedia Appendix 1 Study questionnaire.

Multimedia Appendix 2 Predicted adoption rates of the COVID-19 contact tracing app in Germany by access, ability, and willingness.

Multimedia Appendix 3 Predicted adoption rates of the COVID-19 contact tracing app in Germany by potential to spread SARS-CoV-2.

Multimedia Appendix 4 Predicted adoption rates of the COVID-19 contact tracing app in Germany by potential vulnerability to COVID-19.

Multimedia Appendix 5 Results of a logistic regression of willingness to use the COVID-19 contact tracing app.

## Abbreviations

CHERRIES: Checklist for Reporting Results of Internet E-Surveys
GESIS-DAS: GESIS Data Archive for the Social Sciences
GIP: German Internet Panel
MCS: Mannheim Corona Study
RKI: Robert Koch Institute
