# Supplementary material for: Barriers to the Large-Scale Adoption of a COVID-19 Contact Tracing App in Germany: Survey Study
Source: J Med Internet Res. 2021 Mar 2;23(3):e23362. doi: 10.2196/23362 (PMC7927947; doi:10.2196/23362)
Supplement: Multimedia Appendix 1 [file jmir_v23i3e23362_app1.pdf]

Multimedia Appendix 1. Questionnaire.

Variable	Question text (English translation)	Question text (German original)
	In the following, we ask you several questions about the use of the official Corona-Warn-App that should be available soon. Maybe you have already heard about it through the media.<br><br>The official Corona-Warn-App is made available by the Robert-Koch-Institute (RKI). The RKI is the central institution of the federal government in the area of disease control and prevention. The Corona-Warn-App can help contain the spread of the corona pandemic by informing persons, who have been in contact with someone infected, of a potential infection.<br><br>We aim to find out to what extent the introduction of this app will be effective at containing the corona pandemic. Therefore, we would like to ask you questions about whether you have the necessary devices and knowledge to install and use the app, and whether you would be willing to install and use the app on your smartphone.<br><br>There are no right and wrong answers. We are interested in your experiences and your opinions.	Im Folgenden stellen wir Ihnen ein paar Fragen zur Nutzung der offiziellen Corona-Warn-App, die bald verfügbar sein soll. Vielleicht haben Sie in den Medien bereits davon gehört.<br><br>Die offizielle Corona-Warn-App wird vom Robert-Koch-Institut (RKI) zur Verfügung gestellt. Das RKI ist die zentrale Einrichtung der Bundesregierung auf dem Gebiet der Krankheitsüberwachung und -prävention. Die Corona-Warn-App kann dazu beitragen, die Ausbreitung der Corona-Pandemie einzudämmen, indem sie Kontaktpersonen von Infizierten über eine mögliche Infektion informiert.<br><br>Wir möchten herausfinden, inwieweit die Einführung dieser App effektiv bei der Eindämmung der Corona-Pandemie sein wird. Deshalb wollen wir Ihnen gerne Fragen darüber stellen, ob Sie die nötigen Geräte und Kenntnisse haben, um die App zu installieren und zu nutzen, und ob Sie die App auf Ihrem Smartphone installieren und nutzen möchten.<br><br>Es gibt keine richtigen und falschen Antworten. Wir sind an Ihren Erfahrungen und Ihren Meinungen interessiert.
SCTM002	To be able to use the official Corona-Warn-App you need a smartphone, i.e. a cell phone that has Internet access and on which apps, i.e. additional programs, can be installed.<br><br>**Do you personally use a smartphone?**<br><br>1 Yes, I use a smartphone.<br>2 No, I don’t use a smartphone.<br>3 I use a cell phone but am not sure whether it is a smartphone.	Um die offizielle Corona-Warn-App nutzen zu können, benötigen Sie ein Smartphone, d.h. ein Mobiltelefon, das Internetzugang hat und auf dem Apps, also zusätzliche Programme, installiert werden können.<br><br>**Nutzen Sie persönlich ein Smartphone?**<br><br>1 Ja, ich nutze ein Smartphone.<br>2 Nein, ich nutze kein Smartphone.<br>3 Ich nutze ein Mobiltelefon, bin mir aber nicht sicher, ob das ein Smartphone ist.
SCTM003	If SCTM002 = 1 (if respondent uses a smartphone)<br><br>**Which of the following types best describes your smartphone?**<br>If you use several smartphones, then please answer this question for the smartphone that you use most often.	Wenn SCTM002 = 1 (wenn Befragte/-r Smartphone nutzt)<br><br>**Welcher der folgenden Typen beschreibt Ihr Smartphone am besten?**<br>Wenn Sie mehrere Smartphones nutzen, dann beantworten Sie diese Frage bitte für das Smartphone, das Sie am häufigsten nutzen.

	1 iPhone<br>2 Android Phone<br>3 Something else: [answer field]<br>4 I am not sure.	1 iPhone<br>2 Android Phone<br>3 Etwas anderes, und zwar: [Antwortfeld]<br>4 Ich bin mir nicht sicher.
SCTM004	If SCTM002 = 1 (if respondent uses a smartphone)<br><br>**How often do you carry your smartphone with you when you leave the house?**<br><br>1 Always<br>2 Most of the time<br>3 Sometimes<br>4 Rarely<br>5 Never	Wenn SCTM002 = 1 (wenn Befragte/-r Smartphone nutzt)<br><br>**Wie häufig haben Sie Ihr Smartphone dabei, wenn Sie das Haus verlassen?**<br><br>1 Immer<br>2 Meistens<br>3 Manchmal<br>4 Selten<br>5 Nie
SCTM005	If SCTM001 = 1 (if respondent uses a smartphone)<br><br>You can download the official Corona-Warn-App in the Apple App Store or in the Google Play Store and install it on your smartphone.<br><br>**Do you know how to install an app, i.e. an additional program, on your smartphone?**<br><br>1 Yes<br>2 No<br>3 I am not sure.	Wenn SCTM001 = 1 (wenn Befragte/-r Smartphone nutzt)<br><br>Die offizielle Corona-Warn-App können Sie im Apple App Store oder im Google Play Store herunterladen und auf Ihrem Smartphone installieren.<br><br>**Wissen Sie, wie man eine App, also ein zusätzliches Programm auf Ihrem Smartphone installiert?**<br><br>1 Ja<br>2 Nein<br>3 Ich bin mir nicht sicher.
SCTM006	If SCTM005 = 2 or 3 (if respondent does not know how to install an app or is not sure)<br><br>**Do you know anyone who could help you with installing the Corona-Warn-App on your smartphone, e.g. family, friends, or neighbors?**<br><br>1 Yes<br>2 No<br>3 I am not sure.	Wenn SCTM005 = 2 oder 3 (wenn Befragte/-r nicht weiß wie man eine App installiert oder sich nicht sicher ist)<br><br>**Kennen Sie jemanden, der Ihnen beim Installieren der Corona-Warn-App auf Ihrem Smartphone helfen könnte, z.B. Familie, Freunde oder Nachbarn?**<br><br>1 Ja<br>2 Nein<br>3 Ich bin mir nicht sicher.
SCTM007	If SCTM002 = 1 (if respondent uses a smartphone)<br><br>Once the Corona-Warn-App has been installed, the app will determine which other users of the app are in your proximity. To do this, the app will <u>not</u> access your position, but will use Bluetooth, a technology that transfers data via radio signal between two devices.<br><br>**Do you know how to activate Bluetooth on your smartphone?**	Wenn SCTM002 = 1 (wenn Befragte/-r Smartphone nutzt)<br><br>Sobald die Corona-Warn-App installiert wurde, wird die App feststellen, welche anderen Nutzer der App in Ihrer Nähe sind. Hierfür wird die App <u>nicht</u> auf Ihren Standort zugreifen, sondern Bluetooth nutzen, eine Technologie, die Daten per Funk zwischen zwei Geräten überträgt.<br><br>**Wissen Sie, wie man Bluetooth auf Ihrem Smartphone aktiviert?**

	1 Yes<br>2 No<br>3 I am not sure.	1 Ja<br>2 Nein<br>3 Ich bin mir nicht sicher.
SCTM008	If SCTM007 = 2 or 3 (if respondent does not know how to activate Bluetooth or is not sure)<br><br>**Do you know anyone who could help you with activating Bluetooth on your smartphone, e.g. family, friends or neighbors?**<br><br>1 Yes<br>2 No<br>3 I am not sure.	Wenn SCTM007 = 2 oder 3 (wenn Befragte/-r nicht weiß wie man Bluetooth aktiviert oder sich nicht sicher ist)<br><br>**Kennen Sie jemanden, der Ihnen beim Aktivieren von Bluetooth auf Ihrem Smartphone helfen könnte, z.B. Familie, Freunde oder Nachbarn?**<br><br>1 Ja<br>2 Nein<br>3 Ich bin mir nicht sicher.
SCTM009	If SCTM002 = 1 (if respondent uses a smartphone)<br><br>The Corona-Warn-App will automatically notify you, if you have been in contact with someone who is infected with the coronavirus. In this process, you will not be informed about who the infected person is.<br><br>For this purport, your smartphone exchanges encrypted identification numbers with other smartphones if the smartphones have been in close proximity to each other for a longer period.<br><br>Using the app is free, voluntary and meets the pertinent data protection laws and regulations. The app is meant to be made available in Germany already this month.<br><br>**Would you install the official Corona-Warn-App on your smartphone when it is available?**<br><br>1 Definitely install<br>2 Probably install<br>3 Maybe install, maybe not install<br>4 Probably not install<br>5 Definitely not install	Wenn SCTM002 = 1 (wenn Befragte/-r Smartphone nutzt)<br><br>Die Corona-Warn-App wird Sie automatisch benachrichtigen, falls Sie Kontakt zu jemandem gehabt haben, der mit dem Coronavirus infiziert ist. Dabei werden Sie nicht erfahren, wer die infizierte Person ist.<br><br>Dafür tauscht Ihr Smartphone verschlüsselte Identifikationsnummern mit anderen Smartphones aus, wenn die Smartphones längere Zeit nahe beieinander waren.<br><br>Die Nutzung der App ist kostenlos, freiwillig und entspricht den geltenden Datenschutzgesetzen und -bestimmungen. Sie soll in Deutschland schon diesen Monat zur Verfügung gestellt werden.<br><br>**Würden Sie die offizielle Corona-Warn-App auf Ihrem Smartphone installieren, wenn sie zur Verfügung steht?**<br><br>1 Auf jeden Fall installieren<br>2 Wahrscheinlich installieren<br>3 Vielleicht installieren, vielleicht nicht installieren<br>4 Wahrscheinlich nicht installieren<br>5 Auf keinen Fall installieren
SCTM010	If SCTM002 = 1 (if respondent uses a smartphone) and SCTM009 != 5 (if respondent has not answered "Definitely not install")<br><br>If you might have been infected by a contact person, you will be asked through the Corona-Warn-App to go into precautionary domestic quarantine and to get tested for the virus.	Wenn SCTM002 = 1 (wenn Befragte/-r Smartphone besitzt) und SCTM009 != 5 (wenn Befragte/-r nicht „Auf keinen Fall installieren" angegeben hat)<br><br>Falls Sie sich an einer Kontaktperson infiziert haben könnten, werden Sie über die Corona-Warn-App gebeten, sich vorsorglich in häusliche Quarantäne zu begeben und sich auf das Virus testen zu lassen.

	**Would you comply with the request of the Corona-Warn-App to go into precautionary domestic quarantine?**<br><br>Persons in domestic quarantine must stay at home for one to two weeks and cease contact with other persons completely.<br><br>1 Definitely comply<br>2 Probably comply<br>3 Maybe comply, maybe not comply<br>4 Probably not comply<br>5 Definitely not comply	**Würden Sie der Bitte der Corona-Warn-App nachkommen, sich vorsorglich in häusliche Quarantäne zu begeben?**<br><br>Personen in häuslicher Corona-Quarantäne müssen für ein bis zwei Wochen zu Hause bleiben und den Kontakt zu anderen Personen komplett einstellen.<br><br>1 Auf jeden Fall nachkommen<br>2 Wahrscheinlich nachkommen<br>3 Vielleicht nachkommen, vielleicht nicht nachkommen<br>4 Wahrscheinlich nicht nachkommen<br>5 Auf keinen Fall nachkommen
SCTM011	If SCTM002 = 1 (if respondent uses a smartphone) and SCTM009 != 5 (if respondent has not answered "Definitely not install")<br><br>**Would you comply with the request of the Corona-Warn-App to get tested for the virus?**<br><br>To get tested for the coronavirus, a doctor takes a sample from the upper or deeper respiratory tracts, for example a swab from your nose, mouth or throat or from coughed up secretion, and subsequently sends the sample to a laboratory.<br><br>1 Definitely comply<br>2 Probably comply<br>3 Maybe comply, maybe not comply<br>4 Probably not comply<br>5 Definitely not comply	Wenn SCTM002 = 1 (wenn Befragte/-r Smartphone besitzt) und SCTM009 != 5 (wenn Befragte/-r nicht „Auf keinen Fall installieren" angegeben hat)<br><br>**Würden Sie der Bitte der Corona-Warn-App nachkommen, sich auf das Virus testen zu lassen?**<br><br>Um Sie auf das Coronavirus zu testen, entnimmt ein Arzt/eine Ärztin eine Probe aus den oberen oder tiefen Atemwegen, zum Beispiel als Abstrich aus dem Nasen-, Mund-, Rachenbereich oder dem abgehusteten Sekret und schickt die Probe anschließend in ein Labor.<br><br>1 Auf jeden Fall nachkommen<br>2 Wahrscheinlich nachkommen<br>3 Vielleicht nachkommen, vielleicht nicht nachkommen<br>4 Wahrscheinlich nicht nachkommen<br>5 Auf keinen Fall nachkommen
SCTM012	If SCTM002 = 1 (if respondent uses a smartphone) and SCTM009 != 5 (if respondent has not answered "Definitely not install")<br><br>If you were tested positive for the virus, you can enter the test result into the Corona-Warn-App.<br><br>By doing this, your contact persons will be notified that they might have got infected. Your contact persons are asked to go into precautionary domestic quarantine and get tested for the virus.<br><br>The information that you are the infected person will not be passed on; you thus	Wenn SCTM002 = 1 (wenn Befragte/-r Smartphone besitzt) und SCTM009 != 5 (wenn Befragte/-r nicht „Auf keinen Fall installieren" angegeben hat)<br><br>Wenn Sie positiv auf das Virus getestet wurden, können Sie dies in der Corona-Warn-App eintragen.<br><br>Dadurch werden Ihre Kontaktpersonen benachrichtigt, dass sie sich angesteckt haben könnten. Ihre Kontaktpersonen werden gebeten, sich vorsorglich in häusliche Quarantäne zu begeben und sich auf das Virus testen zu lassen.<br><br>Dass es sich bei der infizierten Person um

	remain anonymous.<br><br>**Would you enter the test result into the Corona-Warn-App if you were tested positive for the virus?**<br><br>1 Definitely enter the result<br>2 Probably enter the result<br>3 Maybe enter the result, maybe not enter the result<br>4 Probably not enter the result<br>5 Definitely not enter the result	Sie handelt, wird nicht weitergegeben; Sie bleiben also anonym.<br><br>**Würden Sie in der Corona-Warn-App eintragen, wenn Sie positiv auf das Virus getestet wurden?**<br><br>1 Auf jeden Fall eintragen<br>2 Wahrscheinlich eintragen<br>3 Vielleicht eintragen, vielleicht nicht eintragen<br>4 Wahrscheinlich nicht eintragen<br>5 Auf keinen Fall eintragen
SCDM004	**Which (occupational) activity is your current main activity?**<br>This concerns your current employment situation in general, independent of whether you have the day off today, for example.<br><br>25 Employee, worker, civil servant (even if you are currently on government-subsidized short-time work or are furloughed)<br>26 Self-employed<br>11 On maternity or parental leave or other leave of absence<br>12 Pupil<br>13 Student<br>14 Pensioner, in early retirement<br>15 Unemployed<br>16 Permanently unable to work<br>17 Homemaker	**Welche (berufliche) Tätigkeit üben Sie derzeit hauptsächlich aus?**<br>Hierbei geht es uns um Ihre aktuelle Erwerbssituation generell, unabhängig davon, ob Sie z.B. heute frei haben.<br><br>25 Angestellte/-r, Arbeiter/-in oder Beamter/-in (auch wenn gerade in Kurzarbeit oder freigestellt)<br>26 Selbstständig<br>11 Im Mutterschafts-, Erziehungsurlaub, Elternzeit oder sonstiger Beurlaubung<br>12 Schüler/-in<br>13 Student/-in<br>14 Rentner/-in, Pensionär/-in, im Vorruhestand<br>15 Arbeitslos<br>16 Dauerhaft erwerbsunfähig<br>17 Hausfrau/Hausmann
SCDM010	If SCDM004 = 25 (if current employment status is employee, worker or civil servant)<br><br>**To what extent are you currently working as an employee, worker or civil servant?**<br>This concerns your contractually agreed and payed work time, not the actual amount of time you are currently working.<br><br>1 I work full-time.<br>2 I work part-time.<br>3 I am on government-subsidized short time, working [answer field] % of a full-time position.<br>4 I am furloughed with pay.<br>5 I am furloughed without pay.	Wenn SCDM004 = 25 (wenn Erwerbsstatus Angestellter, Arbeiter oder Beamter)<br><br>**In welchem Umfang arbeiten Sie derzeit als Angestellte/-r, Arbeiter/-in oder Beamter/-in?**<br>Es geht hier um die vertraglich vereinbarte und entlohnte Arbeitszeit, nicht um die tatsächliche Zeit, die Sie derzeit mit der Arbeit verbringen.<br><br>1 Ich arbeite Vollzeit<br>2 Ich arbeite Teilzeit.<br>3 Ich bin in Kurzarbeit mit [Antwortfeld] % einer Vollzeitstelle.<br>4 Ich bin freigestellt mit Lohnfortzahlung.<br>5 Ich bin freigestellt ohne Lohnfortzahlung.
SCDM011	If SCDM004 = 26 (if current employment status is self-employed)<br><br>**To what extent are you currently working in your self-employed activity?**	Wenn SCDM004 = 26 (wenn Erwerbsstatus Selbstständig)<br><br>**In welchem Umfang arbeiten Sie derzeit in Ihrer selbstständigen Tätigkeit?**

	1 I work more than before the beginning of the corona pandemic.<br>2 I work about the same amount as before the beginning of the corona pandemic.<br>3 I work less than before the beginning of the corona pandemic.<br>4 I currently do not work at all in my self-employed activity.	1 Ich arbeite mehr als vor Beginn der Corona-Pandemie.<br>2 Ich arbeite in etwa gleich viel wie vor der Corona-Pandemie.<br>3 Ich arbeite weniger als vor Beginn der Corona-Pandemie.<br>4 Ich arbeite gerade gar nicht in meiner selbstständigen Tätigkeit.
SCDL012	If (SCDM004 = 25 or SCDM004 = 26) and if (SCDM010 != 4,5 & SCDM010 != 0 & SCDM011 != 4) (if current employment status is employee, worker or civil servant or self-employed and working at the moment)<br><br>**Where do you currently work in your main activity?**<br><br>1 Exclusively on-site at my employer or client<br>2 Predominantly on-site at my employer or client, occasionally from home<br>3 Approximately to the same extent on-site at my employer and from home<br>4 Predominantly from home, occasionally on-site at my employer or client<br>5 Exclusively from home	Wenn (SCDM004 = 25 oder SCDM004 = 26) und wenn (SCDM010 != 4,5 & SCDM010 != 0 & SCDM011 != 4) (wenn Erwerbsstatus Angestellter, Arbeiter oder Beamter oder Selbstständig und aktuell auch arbeitet)<br><br>**Wo arbeiten Sie in Ihrer Haupttätigkeit derzeit?**<br><br>1 Ausschließlich vor Ort beim Arbeitgeber oder Auftraggeber<br>2 Überwiegend vor Ort beim Arbeitgeber oder Auftraggeber, gelegentlich im Homeoffice<br>3 Etwa zu gleichen Teilen vor Ort beim Arbeitgeber und im Homeoffice<br>4 Überwiegend im Homeoffice, gelegentlich beim Arbeitgeber oder Auftraggeber<br>5 Ausschließlich im Homeoffice
SCBM002	**<u>In the last 7 days</u>, how often have you met with friends, relatives or socially with work colleagues?**<br><br>1 Not at all<br>2 Once this week<br>3 Several times this week<br>4 Daily or several times a day<br><br>-99 don’t know	**Wie oft haben Sie sich <u>in den vergangenen 7 Tagen</u> mit Freunden, Verwandten oder privat mit Arbeitskollegen getroffen?**<br><br>1 Gar nicht<br>2 Einmal in dieser Woche<br>3 Mehrmals in dieser Woche<br>4 Täglich oder mehrmals am Tag<br><br>-99 weiß nicht
SCTM001	**Do you suffer from one or more of the following health conditions: overweight, diabetes, high blood pressure, heart or respiratory problems, illnesses of the lung or liver, cancer, or are you immunocompromised?**<br><br>1 Yes<br>2 No	**Leiden Sie unter einem oder mehreren der folgenden Gesundheitsprobleme: Übergewicht, Diabetes, Bluthochdruck, Herz- oder Atemprobleme, Lunge-, Leber- oder Krebserkrankungen oder einem geschwächten Immunsystem?**<br><br>1 Ja<br>2 Nein
AA43404	**Please provide your year of birth.**<br><br>**Year of birth:**<br>[answer field]	**Bitte geben Sie ihr Geburtsjahr an.**<br><br>**Geburtsjahr:**<br>[Antwortfeld]
