# Supplementary material for: Barriers to the Large-Scale Adoption of a COVID-19 Contact Tracing App in Germany: Survey Study
Source: J Med Internet Res. 2021 Mar 2;23(3):e23362. doi: 10.2196/23362 (PMC7927947; doi:10.2196/23362)
Supplement: Multimedia Appendix 2 [file jmir_v23i3e23362_app2.pdf]

Multimedia Appendix 2. Predicted adoption rates of the COVID-19 contact tracing app in Germany by access, ability, and willingness.

**Access**	%	CI (%)	*n*
Uses smartphone	91.8	[90.5; 93.0]	2,836
Has compatible operating system	88.5	[86.9; 89.9]	2,732
Uses smartphone outside the house	85.0	[83.3; 86.6]	2,626
Total access	82.6	[80.8; 84.2]	2,549
**+Ability**			
Can install apps on a smartphone	81.3	[79.5; 83.0]	2,511
Can activate Bluetooth on a smartphone	81.8	[80.0; 83.5]	2,525
Total ability	81.0	[79.1; 82.7]	2,500
**+Willingness**			
Willing to install the app	37.9	[35.8; 40.0]	1,170
Willing to go into quarantine if exposed to infected person	34.9	[32.9; 37.0]	1,079
Willing to get tested if exposed to infected person	37.3	[35.3; 39.5]	1,153
Willing to enter test result into app if tested positive	37.6	[35.6; 39.8]	1,162
Total willingness	34.7	[32.7; 36.8]	1,070

Note. N=3,276. %=weighted proportions. CI=95% confidence intervals. *n*=weighted counts.
