# Supplementary material for: Barriers to the Large-Scale Adoption of a COVID-19 Contact Tracing App in Germany: Survey Study
Source: J Med Internet Res. 2021 Mar 2;23(3):e23362. doi: 10.2196/23362 (PMC7927947; doi:10.2196/23362)
Supplement: Multimedia Appendix 3 [file jmir_v23i3e23362_app3.pdf]

Multimedia Appendix 3. Predicted adoption rates of the COVID-19 contact tracing app in Germany by potential to spread SARS-CoV-2.

	Has access to the app		Able to use the app		Willing to use the app	
	%	CI (%)	%	CI (%)	%	CI (%)
Total	82.6	[80.8; 84.2]	81.0	[79.1; 82.7]	34.7	[32.7; 36.8]
Met socially several times last week, worked full-time outside home	91.8^A	[87.3; 94.8]	90.7^D	[85.9; 93.9]	31.0^G	[25.8; 36.7]
Met socially several times last week, did not work full-time outside home	83.0^B	[79.9; 85.7]	81.9^E	[78.8; 84.7]	37.0^G	[33.5; 40.7]
Met socially once or less last week, worked full-time outside home	85.9^B	[81.5; 89.5]	84.4^E	[79.9; 88.0]	32.6^G	[28.0; 37.6]
Met socially once or less last week, did not work full-time outside home	77.4^C	[74.1; 80.4]	75.2^F	[71.7; 78.3]	35.2^G	[31.8; 38.7]
N	3,267		3,267		3,266	

Note. %=weighted proportions. CI=95% confidence intervals. Values with different letters within a column are statistically significant (Chi-2 test) at $P < .05$.
