# Supplementary material for: Barriers to the Large-Scale Adoption of a COVID-19 Contact Tracing App in Germany: Survey Study
Source: J Med Internet Res. 2021 Mar 2;23(3):e23362. doi: 10.2196/23362 (PMC7927947; doi:10.2196/23362)
Supplement: Multimedia Appendix 4 [file jmir_v23i3e23362_app4.pdf]

Multimedia Appendix 4. Predicted adoption rates of the COVID-19 contact tracing app in Germany by potential vulnerability to COVID-19.

	Has access to the app		Able to use the app		Willing to use the app	
	%	CI (%)	%	CI (%)	%	CI (%)
Total	82.6	[80.8; 84.2]	81.0	[79.1; 82.7]	34.7	[32.7; 36.8]
Age 60-77, with at-risk health conditions	62.4[A]	[57.3; 67.4]	60.6[D]	[55.4; 65.6]	35.9[G,H]	[31.1; 40.9]
Age 60-77, without at-risk health conditions	63.2[A]	[56.5; 69.5]	59.7[D]	[52.9; 66.1]	34.7[G,H]	[28.7; 41.3]
Age 18-59, with at-risk health conditions	87.1[B]	[83.2; 90.2]	85.8[E]	[81.9; 89.0]	41.3[G]	[37.0; 45.7]
Age 18-59, without at-risk health conditions	91.8[C]	[89.8; 93.4]	90.6[F]	[88.5; 92.3]	30.6[H]	[27.8; 33.4]
N	3,270		3,270		3,269	

Note. %=weighted proportions. CI=95% confidence intervals. Values with different letters within a column are statistically significant (Chi-2 test) at $P < .05$.
