# Supplementary material for: Barriers to the Large-Scale Adoption of a COVID-19 Contact Tracing App in Germany: Survey Study
Source: J Med Internet Res. 2021 Mar 2;23(3):e23362. doi: 10.2196/23362 (PMC7927947; doi:10.2196/23362)
Supplement: Multimedia Appendix 5 [file jmir_v23i3e23362_app5.pdf]

Multimedia Appendix 5. Results of a logistic regression of willingness to use the COVID-19 contact tracing app.

	Willing to use the app	
	OR	SE
Interviewed after app launch	1.092	(0.103)
(Ref: Before app launch)		
Age	1.005	(0.004)
Gender (Ref: Male)	0.856	(0.084)
Highest level of education (Ref: Low)		
Intermediate	1.034	(0.153)
High	1.121	(0.171)
Household net income (monthly)		
(Ref: €0-1,999)		
€2,000-2,999	0.930	(0.144)
€3,000-3,999	1.254	(0.206)
€4,000+	1.691**	(0.273)
Household size (Ref: 1)		
2	0.951	(0.143)
3+	0.863	(0.133)
Federal state		
(Ref: Baden-Württemberg)		
Bayern	0.971	(0.163)
Berlin	1.615	(0.444)
Brandenburg	0.766	(0.229)
Bremen	1.116	(0.596)
Hamburg	1.033	(0.377)
Hessen	1.473*	(0.289)
Mecklenburg-Vorpommern	1.018	(0.401)
Niedersachsen	1.310	(0.250)
Nordrhein-Westfalen	1.721**	(0.279)
Rheinland-Pfalz	1.173	(0.323)
Saarland	1.159	(0.479)
Sachsen	0.731	(0.166)
Sachsen-Anhalt	0.583	(0.222)
Schleswig-Holstein	1.608	(0.493)
Thüringen	0.761	(0.246)
Vote at last general election		
(Ref: CDU/CSU)		
SPD	0.990	(0.148)
FDP	0.900	(0.162)

Bündnis 90/Die Grünen	1.064	(0.146)
Die Linke	0.673	(0.143)
AfD	0.494**	(0.104)
Other party	0.368**	(0.115)
Did not vote	0.301***	(0.066)
Not eligible to vote	0.445**	(0.130)
N	3,274	
Pseudo R-squared	0.065	

Note. *P < .05, **P < .01, ***P < 0.001.

OR=odds ratio. SE=robust standard error.
